# Anaesthesia for caesarean section in the presence of multivalvular heart disease and severe pulmonary hypertension: a case report

**DOI:** 10.1186/1757-1626-2-9383

**Published:** 2009-12-22

**Authors:** Demet Coskun, Ahmet Mahli, Sibel Korkmaz, Figen S Demir, Gozde Karaca Inan, Dilek Erer, M Emin Ozdogan

**Affiliations:** 1 Department of Anesthesiology, Gazi University Faculty of Medicine, Besevler, Ankara, Turkey; 2 Department of Cardiovascular Surgery, Gazi University Faculty of Medicine, Besevler, Ankara, Turkey

## Abstract

**Introduction:**

Pulmonary hypertension is a rare condition and in combination with pregnancy, it can result in high maternal mortality. Mitral stenosis is one of the complicated cardiac diseases that may occur during pregnancy. In this report, we describe our management of such a case, which was even more difficult in combination with pulmonary hypertension, mitral stenosis, and aortic and tricuspid valve insufficiency requiring emergency caesarean section under general anaesthesia.

**Case presentation:**

A 29-year-old primiparae was presented to the anaesthetic department for an urgent caesarean section with a diagnosis of severe pulmonary hypertension in combination with mitral stenosis. The patient was hospitalized prepartum and received oxygen therapy and anticoagulation with heparin. The patient was monitored during labour and delivery with oximetry and arterial and central venous pressure line. Pulmonary arterial lines were not used due to an increased risk and questionable usefulness. Echocardiography revealed a systolic pulmonary arterial pressure of 75 mmHg, and mitral stenosis, aortic and tricuspid valve insufficiency.

We decided to proceed under general anaesthesia. Anaesthesia was induced with etomidate, and succinylcholine. Dopamine and nitroglycerin infusion was preoperatively started and infusion was also preoperatively continued. Hemodynamic parameters were stable during delivery. Neonatal weight and apgar score were satisfactory. After the delivery of a healthy baby, oxytocin was administered. Surgery was completed uneventfully. During the postoperative period, the patient received furosemide and morphine. As the arterial blood gas analyses were stable and the chest-ray was normal, the patient was extubated postoperatively in the second hour in ICU.

**Conclusion:**

Patients with significant multivalvular heart disease require careful preoperative, multidisciplinary assessment and anesthetic planning before delivery in order to optimize cardiac function during the peripartum period and make informed decisions regarding the mode of delivery and anaesthetic technique.

## Introduction

Pulmonary hypertension is a rare condition and in combination with pregnancy, it can result in high maternal mortality. Mitral stenosis is one of the complicated cardiac diseases that may occur during pregnancy. Furthermore, despite the improvements in medical, obstetric, anaesthetic, and intensive care, mortality rates still remain disappointingly high [1,2].

In this report, we describe our management of such a case, which was even more difficult in combination with pulmonary hypertension, mitral stenosis, and aortic and tricuspid valve insufficiency requiring urgent Caesarean section under general anaesthesia.

## Case report

A 29-year-old Turkish primigravid parturient with a height of 160 cm and a weight of 80 kg was presented to the anaesthetic department for an urgent Caesarean section with a diagnosis of severe pulmonary hypertension and associated multivalvular disease. At 35 weeks' gestation, she experienced palpitations, shortness of breath, dizziness, and dyspnea so she was referred for cardiology consultation. During examination, a systolic murmur (grade 2/6) was present in all auscultation areas. There was some evidence of pulmonary edema in the chest X-ray; although she did not have hepatomegaly, there was 1+/1+ peripheral edema. The electrocardiogram showed a sinus rhythm of 95 beats.min^-1 ^with a normal axis, borderline right ventricular hypertrophy and an arterial blood pressure of 118/62 mmHg. She underwent echocardiography that revealed severe pulmonary hypertension with a systolic pulmonary artery pressure of 75 mmHg and an associated mitral stenosis with a mitral valve area of 1.3 cm^2^. The right ventricle was dilated; the echocardiography demonstrated a mild mitral regurgitation, moderate-severe aortic regurgitation and moderate tricuspid regurgitation. Haematological and biochemical investigations were within normal limits except an Hb value of 9,42 gr.L^-1^. She was hospitalized in coronary intensive care unit and treated with diuretics. At home, she was anticoagulated with low molecular heparin (Clexane^®^), whereas cardiologists started heparin infusion at a rate of 1000 U.hr^-1 ^when she was hospitalized. Serial ultrasound and cardiotocography tracings confirmed that fetal growth was normal. At 36 weeks' gestation when active labour began, it was decided that she should undergo Caesarean section because induction of labour was considered inappropriate.

**Figure 1:**
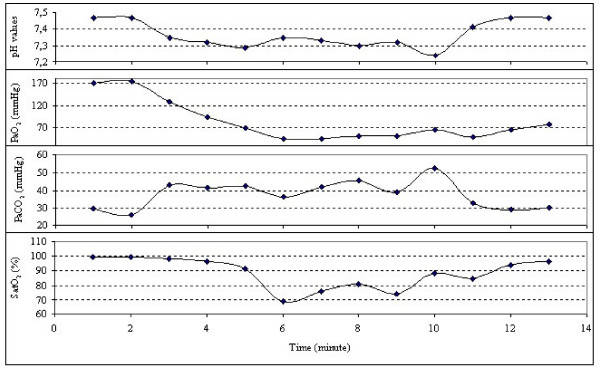
**Perioperative blood gas analyses**.

In the operating room, non-invasive arterial pressure monitoring, 6-lead ECG with ST-segment analysis, and pulse oximetry were applied. She was tachycardic and tachypneic. Preoperative and perioperative haemodynamic and respiratory parameters were recorded (Table 1). Preoxygenation and cricoid pressure were applied; general anaesthesia was induced with etomidate 0.3 mg.kg^-1^, succinylcholine 1 mg.kg^-1^, and lidocaine 1 mg.kg^-1^. The patient was intubated and ventilated with 100% oxygen until delivery. Arterial and central venous catheterization was attemted, and than invasive arterial pressure and central venous pressure were monitored and arterial blood gas analyses were obtained every two minutes perioperatively (Figure 1). Infusions of glyceryl trinitrate and dopamine were started preoperatively and continued perioperatively. Central venous pressure was maintained at 5-10 mmHg throughout the operation. After a healthy 2450 g baby was delivered with an Apgar score of 9 at the 1^st ^and 5^th ^minutes and an infusion of oxytocin (20 U for more than 2 hours) was started. Anaesthesia was maintained with isoflurane, and 50% nitrous oxide in oxygen and 0,05 mg.kg^-1 ^vecuronium, and 100 μg IV fentanyl were administered. During delivery, the patient's pulmonary edema increased, and a decrease in oxygen saturation was observed. Pulmonary edema during delivery was rapidly resolved after diuretic administration. Surgery was completed uneventfully. After the operation, the patient was admitted to the intensive care unit where artificial ventilation and continuous monitorization were continued. During the postoperative period, the patient was sedated using an infusion of propofol and received morphine, furosemide, and glyceryl trinitrate at adequate doses. When the arterial blood gas analyses and the chest-X-ray were normal, the patient was extubated postoperatively in the second hour in the intensive care unit. She was discharged home in good condition after one week following the operation and was advised to undergo cardiac surgery.

**Table 1:** Perioperative heamodynamic and respiratory parameters.

	HR (BPM)	SAP (mmHg)	DAP (mmHg)	MAP (mmHg)	sPO_2_ (%)
**Control**	105	111	59	78	97

**Induction 1^st ^min**	108	107	43	65	96

**Delivery 1^st ^min**	115	171	89	110	95

**Delivery 5^th ^min**	109	116	55	69	96

**Skin closure**	103	106	43	63	96

**End of operation**	109	133	66	88	96

**Intensive care unit (under sedation)**	98	80	56	63	96

**Extubation**	112	151	78	108	97

HR, Heart rate; SAP, Systolic arterial pressure; DAP, Diastolic arterial pressure; MAP, Mean arterial pressure; sPO_2_, Saturation of peripheral oxygen

## Discussion

Cardiovascular stress owing to pregnancy, labour, delivery, and the postdelivery period induce different degrees of cardiac failure in every cardiac patient, and concomitant cardiac medication and therapeutic anticoagulation interfere with the anaesthetic management [3]. Adequate cardiovascular invasive monitoring is essential and should be administered and maintained in the postpartum period with the same criteria that reduce morbidity and mortality in cardiac patients undergoing general surgery [3,4].

In our case, standard vascular access included a radial artery catheter, an internal jugular central venous line, and a large venous catheter for rapid fluid infusion. We preferred not to attempt to insert central venous and arterial catheters until the patient was intubated as we considered that this procedure would be too stressful for this anxious patient and was likely to result in a further increase in heart rate.

This patient received her diagnosis late in pregnancy, beyond the time at which a therapeutic termination could have been performed. She was managed with a multidisciplinary approach, and her care included cardiologists and obstetricians but she was not consulted to anesthesiologists until delivery. But still, general anaesthesia and Caesarean section were successfully performed. She received aggressive anticoagulation as deep venous thrombosis and pulmonary embolism are important causes of postpartum mortality in patients with pulmonary arterial hypertension. Caesarean delivery was planned at 36 weeks' gestation to maximize fetal lung maturation and to avoid deterioration in maternal cardiac status.

There are no controlled studies examining the best type of anaesthetic technique in these patients, and guidelines and standards are lacking. Although no curative agent has been identified, the practitioners' knowledge of the existing treatment options, pathophysiology, and the implications of various anaesthetic agents and techniques is required to ensure the highest level of patient safety and care [5]. Experts recommend individualizing the anaesthetic management according to the parturient's cardiovascular status and general pathophysiological concepts [3]. Some authors have described the use of general anaesthesia with good maternal outcome [6,7]. However, others have reported increased pulmonary arterial pressure during laryngoscopy and tracheal intubation; moreover, adverse effects of positive-pressure ventilation on venous return may ultimately lead to cardiac failure [8,9].

As this patient was anticoagulated aggressively, general anaesthesia was preferred. Opioid-based techniques are recommended for anaesthesia in patients with valvular disease as they have a minimally depressive action on the cardiovascular system and provide excellent analgesia. But we were concerned that use of opioids in induction could result in respiratory depression of neonate, so fentanyl was not administered until delivery. However, to avoid an increase in systemic and pulmonary pressures resulting from laryngoscopy and tracheal intubation, lidocaine was administered and glyceryl trinitrate infusion was started preoperatively and continued postoperatively. Also, depths of analgesia and anaesthesia levels were maintained adequately throughout the surgery in order to avoid tachycardia and hypertension. We tried to provide lower peak inspiratory pressures to avoid these adverse effects of artificial ventilation.

A systolic pulmonary artery pressure of above 50 mmHg is associated with cardiac complications during pregnancy as functional status worsens more rapidly in pregnant than in non-pregnant patients with mitral valve stenosis. Cardiac decompensation and pulmonary edema may occur in pregnant women with overt or silent mitral valve stenosis during the second or third trimester. Fluid restriction, diuretics, and control of atrial fibrillation are basic measures that can prevent pulmonary congestion [1].

The postpartum period is the most critical period for acute pulmonary hypertension decompensations [4]. Symptomatic therapy during the postpartum period may include inhaled nitric oxide and epoprostenol infusion or inhaled iloprost [1,7,10]. For women with unexpected primary pulmonary hypertension who need emergency Cesarean section, inhaled nitric oxide is used [11]. In this case, pulmonary edema that occurred after delivery was resolved with diuretics with no need of using inhaled nitric oxide or other pulmonary vasodilators.

## Conclusion

Patients with significant multivalvular heart disease require careful preoperative, multidisciplinary assessment and anaesthetic planning before delivery in order to optimize cardiac function during the peripartum period and make informed decisions regarding the mode of delivery and anaesthetic technique. Particularly the period after delivery carries a high risk of maternal death. Therefore, prolonged intensive care for both pre and postpartum periods is essential.

## Consent

Written informed consent was obtained from the patient for publication of this case report and accompanying images. A copy of the written consent is available for review by the Editor-in-Chief of this journal.

## Competing interests

The authors declare that they have no competing interests.

## Authors' contributions

DC and AM presented the case history, researched the topic and helped draft the manuscript. SK, SFD, DE and MEO reviewed the literature and drafted the manuscript. All authors read and approved the final manuscript.
